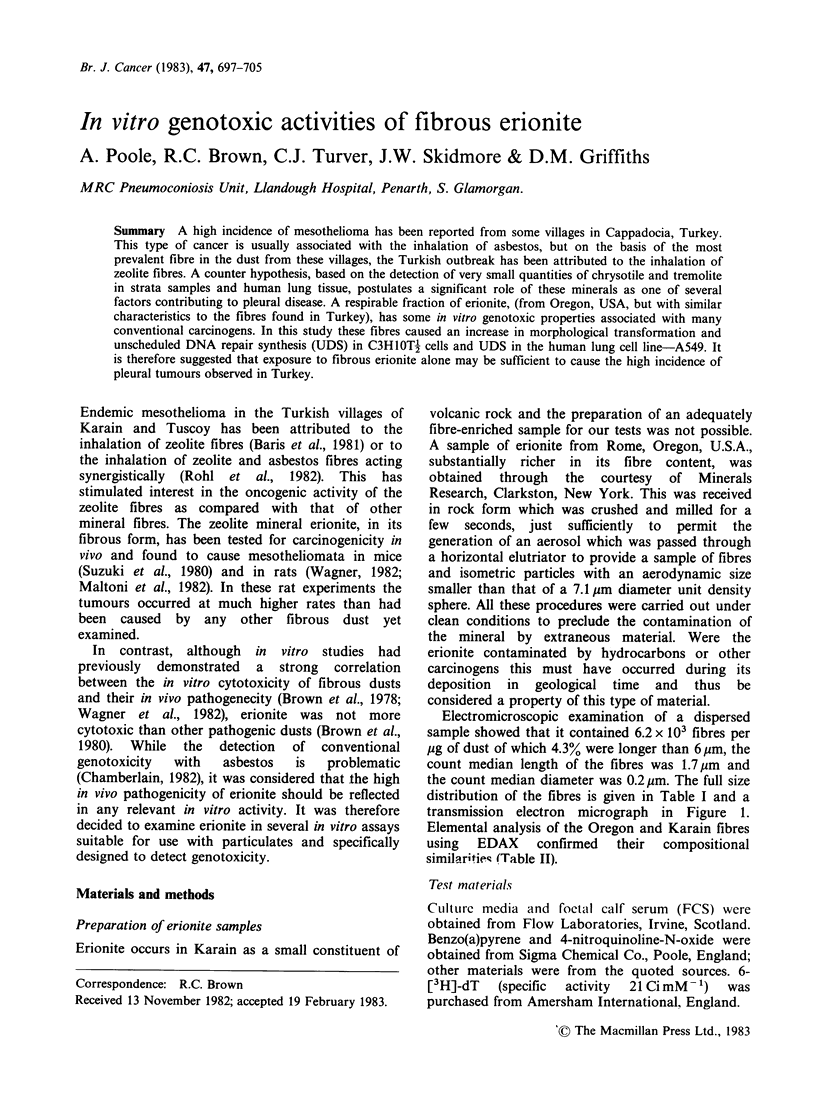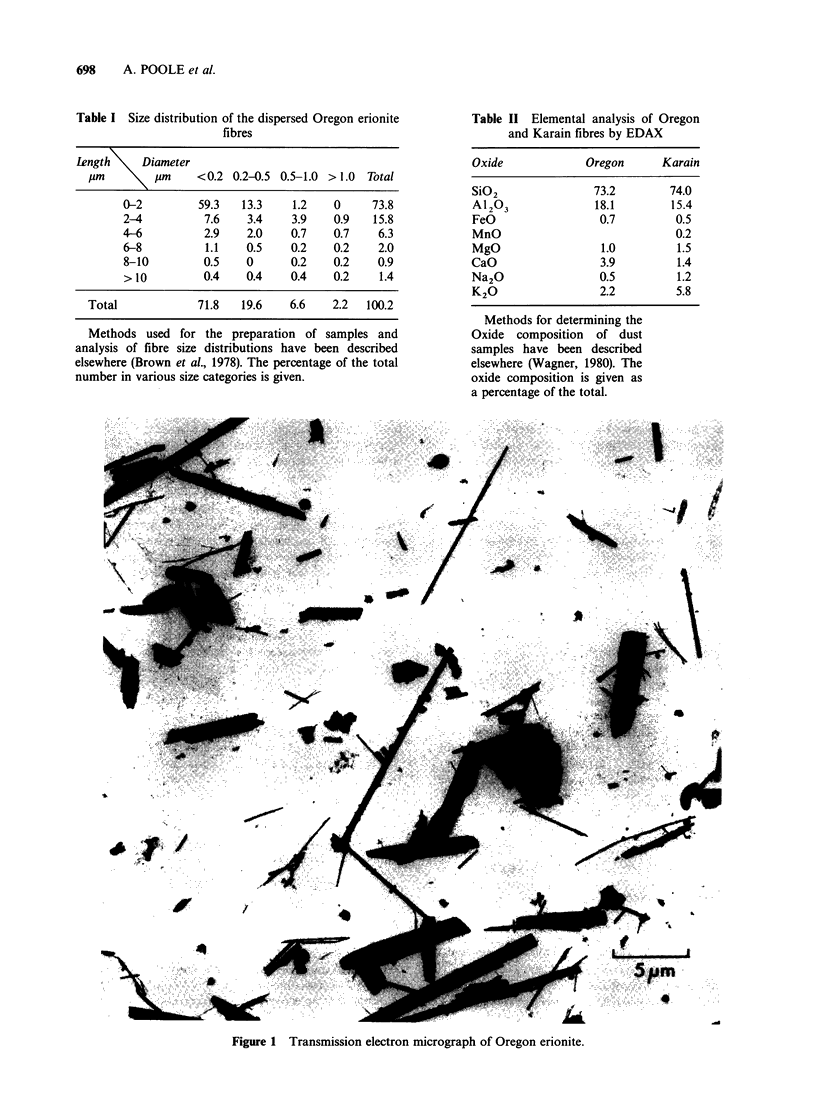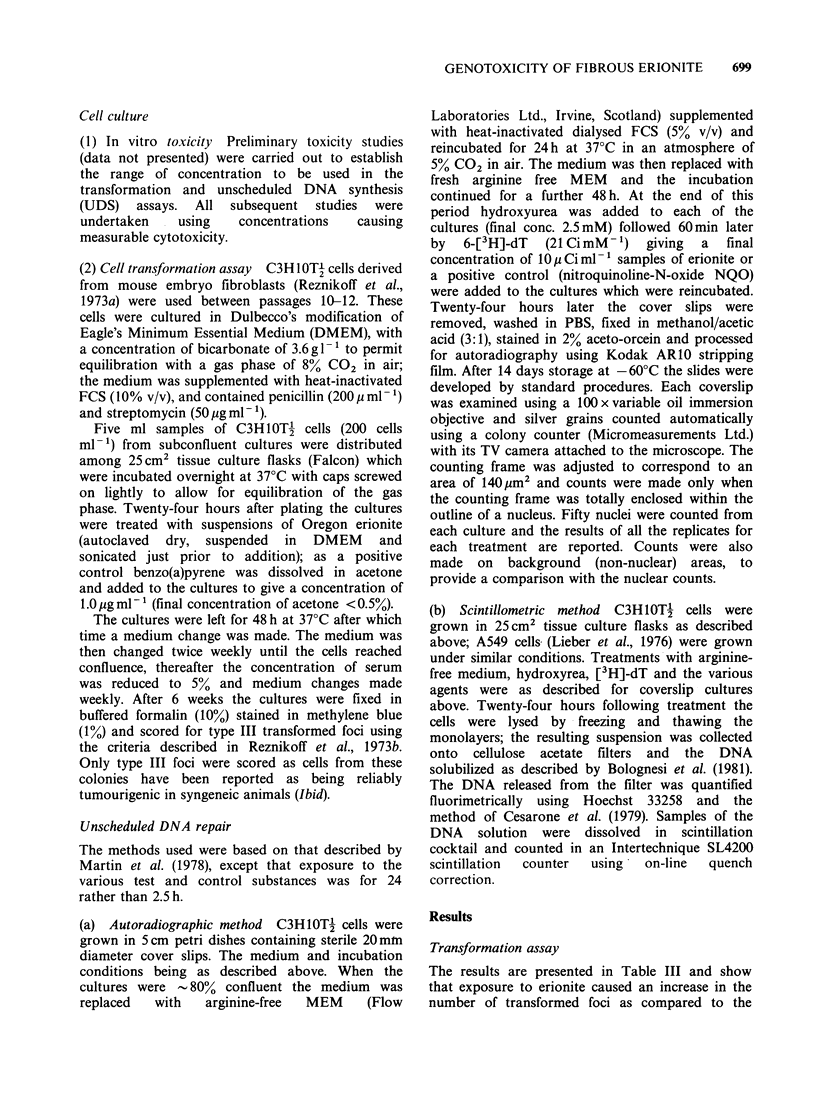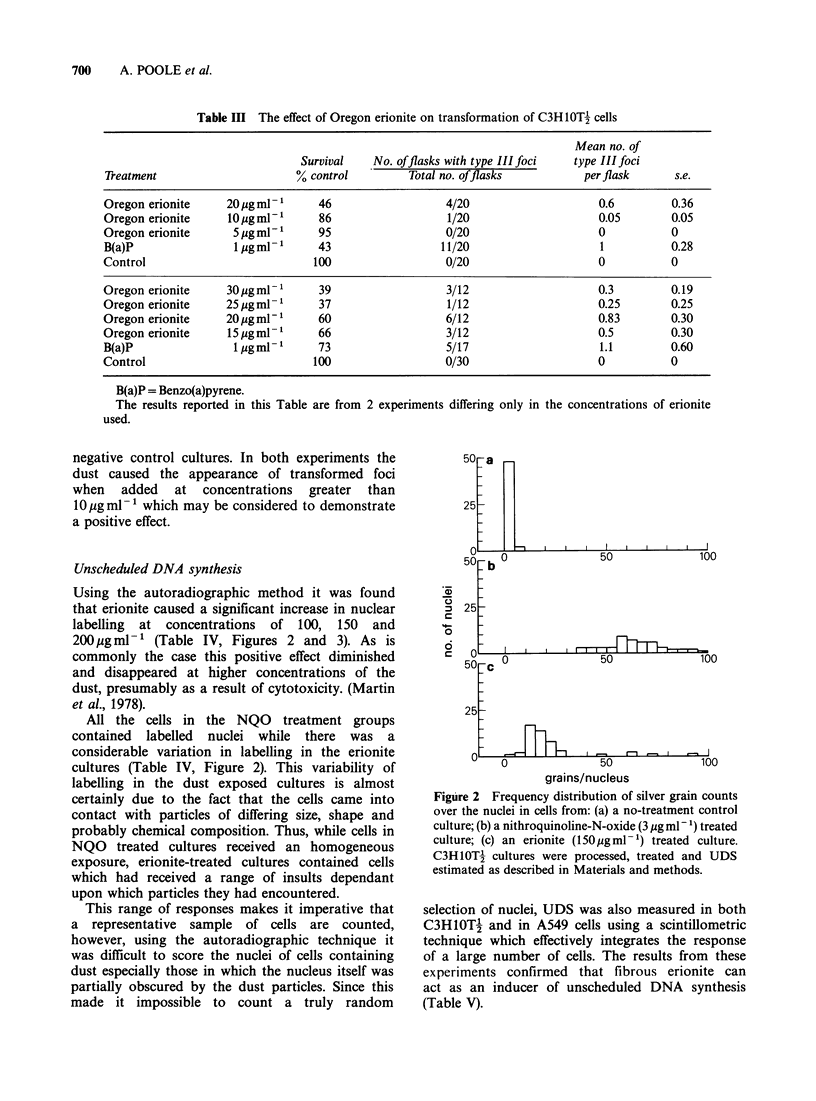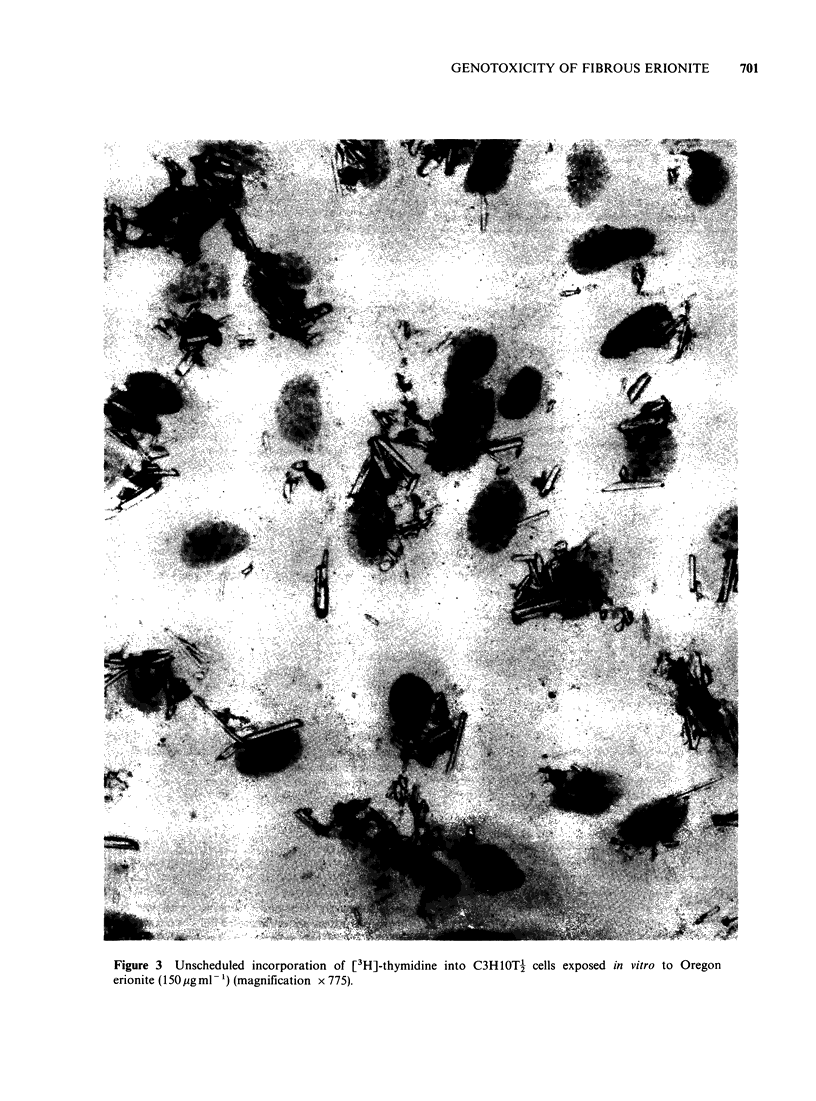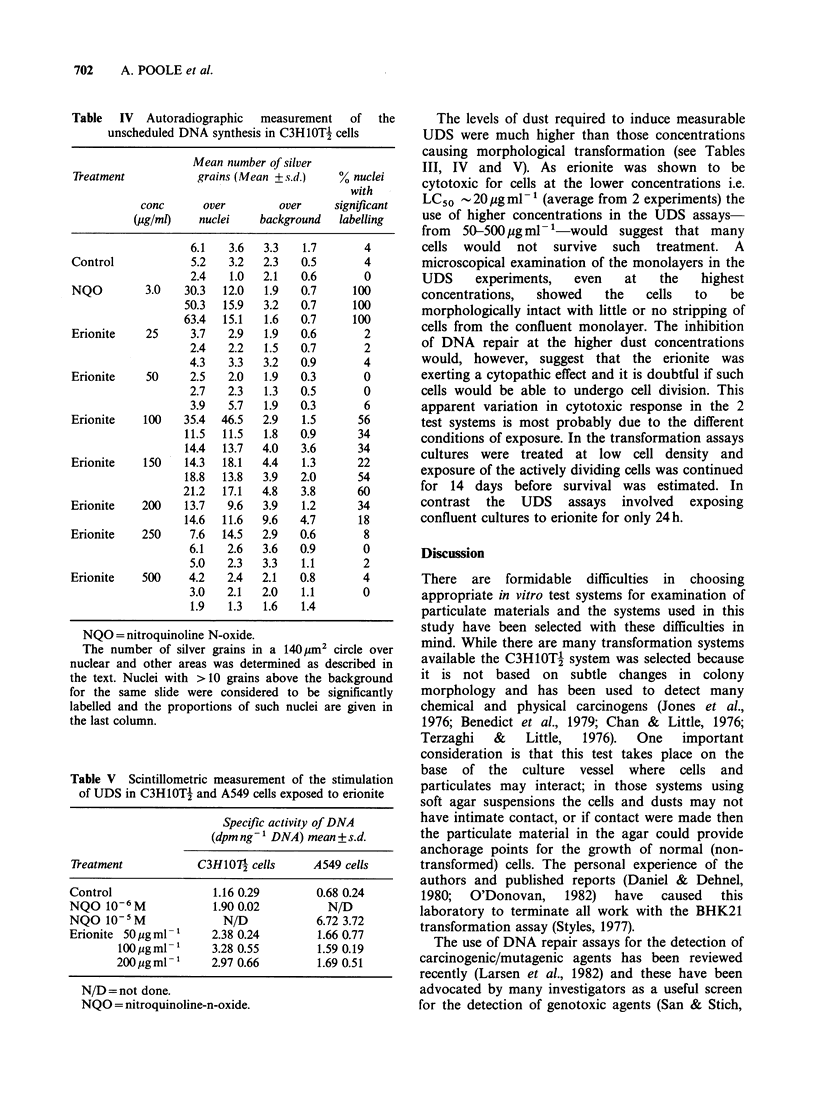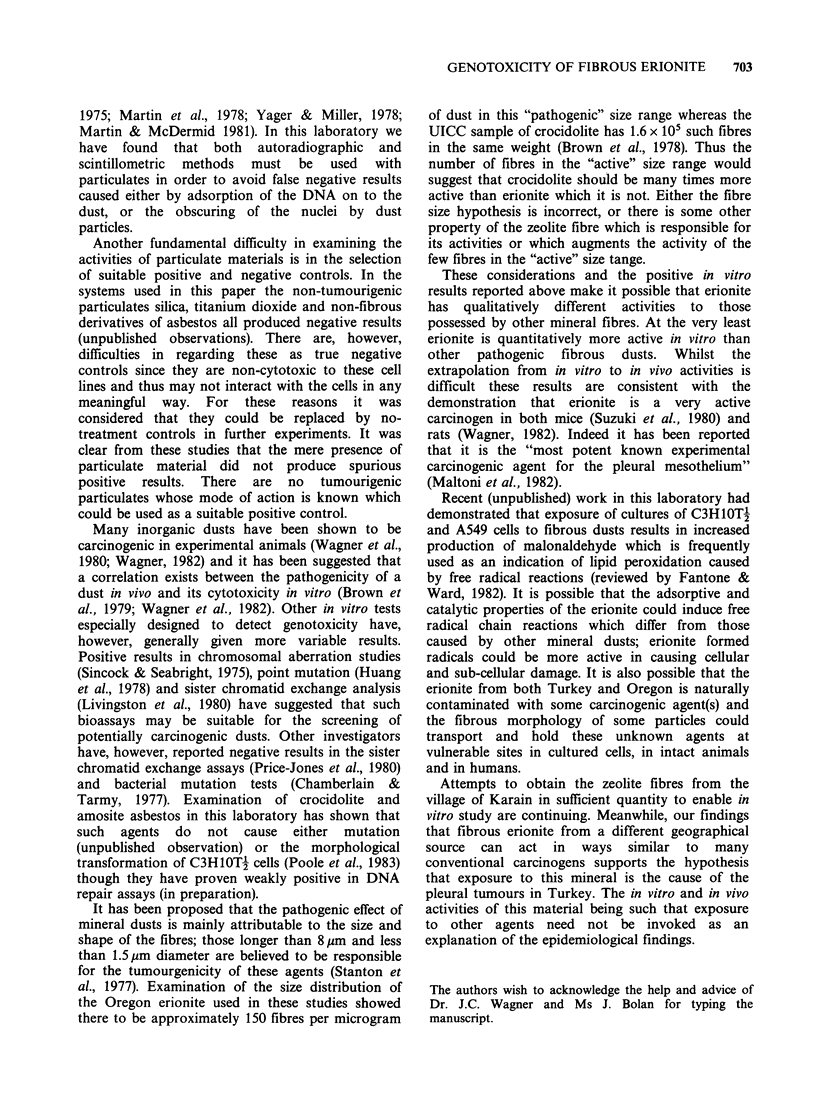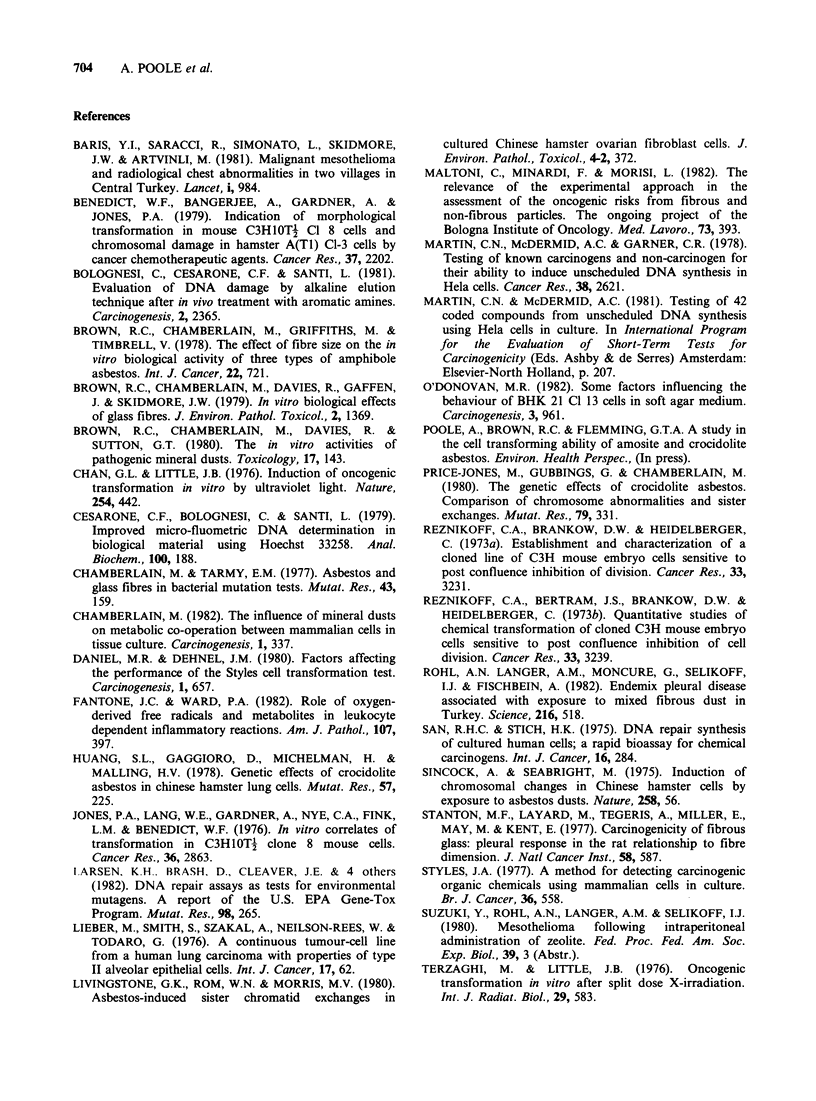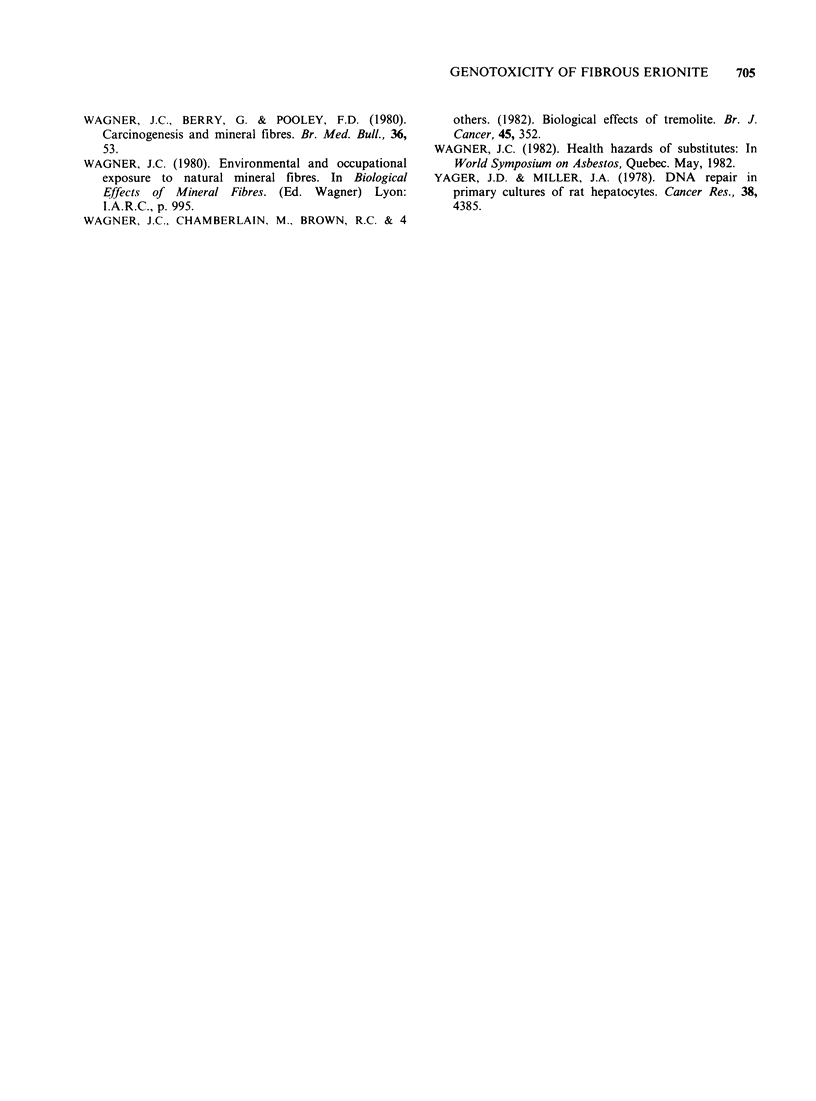# In vitro genotoxic activities of fibrous erionite.

**DOI:** 10.1038/bjc.1983.108

**Published:** 1983-05

**Authors:** A. Poole, R. C. Brown, C. J. Turver, J. W. Skidmore, D. M. Griffiths

## Abstract

**Images:**


*Br. J. Cancer* (1983), **47**, 697–705

### *In vitro* genotoxic activities of fibrous erionite

A. Poole, R.C. Brown, C.J. Turver, J.W. Skidmore & D.M. Griffiths

*MRC Pneumoconiosis Unit, Llandough Hospital, Penarth, S. Glamorgan.*

**Summary** A high incidence of mesothelioma has been reported from some villages in Cappadocia, Turkey. This type of cancer is usually associated with the inhalation of asbestos, but on the basis of the most prevalent fibre in the dust from these villages, the Turkish outbreak has been attributed to the inhalation of zeolite fibres. A counter hypothesis, based on the detection of very small quantities of chrysotile and tremolite in strata samples and human lung tissue, postulates a significant role of these minerals as one of several factors contributing to pleural disease. A respirable fraction of erionite, (from Oregon, USA, but with similar characteristics to the fibres found in Turkey), has some *in vitro* genotoxic properties associated with many conventional carcinogens. In this study these fibres caused an increase in morphological transformation and unscheduled DNA repair synthesis (UDS) in C3H10T½ cells and UDS in the human lung cell line—A549. It is therefore suggested that exposure to fibrous erionite alone may be sufficient to cause the high incidence of pleural tumours observed in Turkey.

Endemic mesothelioma in the Turkish villages of Karain and Tuscoy has been attributed to the inhalation of zeolite fibres (Baris *et al.*, 1981) or to the inhalation of zeolite and asbestos fibres acting synergistically (Rohl *et al.*, 1982). This has stimulated interest in the oncogenic activity of the zeolite fibres as compared with that of other mineral fibres. The zeolite mineral erionite, in its fibrous form, has been tested for carcinogenicity *in vivo* and found to cause mesotheliomata in mice (Suzuki *et al.*, 1980) and in rats (Wagner, 1982; Maltoni *et al.*, 1982). In these rat experiments the tumours occurred at much higher rates than had been caused by any other fibrous dust yet examined.

In contrast, although *in vitro* studies had previously demonstrated a strong correlation between the *in vitro* cytotoxicity of fibrous dusts and their *in vivo* pathogenecity (Brown *et al.*, 1978; Wagner *et al.*, 1982), erionite was not more cytotoxic than other pathogenic dusts (Brown *et al.*, 1980). While the detection of conventional genotoxicity with asbestos is problematic (Chamberlain, 1982), it was considered that the high *in vivo* pathogenicity of erionite should be reflected in any relevant *in vitro* activity. It was therefore decided to examine erionite in several *in vitro* assays suitable for use with particulates and specifically designed to detect genotoxicity.

### Materials and methods

### *Preparation of erionite samples*

Erionite occurs in Karain as a small constituent of volcanic rock and the preparation of an adequately fibre-enriched sample for our tests was not possible. A sample of erionite from Rome, Oregon, U.S.A., substantially richer in its fibre content, was obtained through the courtesy of Minerals Research, Clarkston, New York. This was received in rock form which was crushed and milled for a few seconds, just sufficiently to permit the generation of an aerosol which was passed through a horizontal elutriator to provide a sample of fibres and isometric particles with an aerodynamic size smaller than that of a 7.1 $\mu$m diameter unit density sphere. All these procedures were carried out under clean conditions to preclude the contamination of the mineral by extraneous material. Were the erionite contaminated by hydrocarbons or other carcinogens this must have occurred during its deposition in geological time and thus be considered a property of this type of material.

Electromicroscopic examination of a dispersed sample showed that it contained $6.2 \times 10^3$ fibres per $\mu$g of dust of which 4.3% were longer than 6 $\mu$m, the count median length of the fibres was 1.7 $\mu$m and the count median diameter was 0.2 $\mu$m. The full size distribution of the fibres is given in Table I and a transmission electron micrograph in Figure 1. Elemental analysis of the Oregon and Karain fibres using EDAX confirmed their compositional similarities (Table II).

### *Test materials*

Culture media and foetal calf serum (FCS) were obtained from Flow Laboratories, Irvine, Scotland. Benzo(a)pyrene and 4-nitroquinoline-N-oxide were obtained from Sigma Chemical Co., Poole, England; other materials were from the quoted sources. 6-[$^3$H]-dT (specific activity 21 Ci mM$^{-1}$) was purchased from Amersham International, England.

Correspondence: R.C. Brown
Received 13 November 1982; accepted 19 February 1983.

© The Macmillan Press Ltd., 1983

**Table I** Size distribution of the dispersed Oregon erionite fibres

| *Length* μm \ *Diameter* μm | <0.2 | 0.2–0.5 | 0.5–1.0 | >1.0 | *Total* |
|---|---|---|---|---|---|
| 0–2 | 59.3 | 13.3 | 1.2 | 0 | 73.8 |
| 2–4 | 7.6 | 3.4 | 3.9 | 0.9 | 15.8 |
| 4–6 | 2.9 | 2.0 | 0.7 | 0.7 | 6.3 |
| 6–8 | 1.1 | 0.5 | 0.2 | 0.2 | 2.0 |
| 8–10 | 0.5 | 0 | 0.2 | 0.2 | 0.9 |
| >10 | 0.4 | 0.4 | 0.4 | 0.2 | 1.4 |
| Total | 71.8 | 19.6 | 6.6 | 2.2 | 100.2 |

Methods used for the preparation of samples and analysis of fibre size distributions have been described elsewhere (Brown *et al.*, 1978). The percentage of the total number in various size categories is given.

**Table II** Elemental analysis of Oregon and Karain fibres by EDAX

| *Oxide* | *Oregon* | *Karain* |
|---|---|---|
| $SiO_2$ | 73.2 | 74.0 |
| $Al_2O_3$ | 18.1 | 15.4 |
| FeO | 0.7 | 0.5 |
| MnO | | 0.2 |
| MgO | 1.0 | 1.5 |
| CaO | 3.9 | 1.4 |
| $Na_2O$ | 0.5 | 1.2 |
| $K_2O$ | 2.2 | 5.8 |

Methods for determining the Oxide composition of dust samples have been described elsewhere (Wagner, 1980). The oxide composition is given as a percentage of the total.

**Figure 1** Transmission electron micrograph of Oregon erionite.

*Cell culture*

(1) In vitro *toxicity* Preliminary toxicity studies (data not presented) were carried out to establish the range of concentration to be used in the transformation and unscheduled DNA synthesis (UDS) assays. All subsequent studies were undertaken using concentrations causing measurable cytotoxicity.

(2) *Cell transformation assay* C3H10T$\frac{1}{2}$ cells derived from mouse embryo fibroblasts (Reznikoff *et al*., 1973*a*) were used between passages 10–12. These cells were cultured in Dulbecco's modification of Eagle's Minimum Essential Medium (DMEM), with a concentration of bicarbonate of 3.6 g l$^{-1}$ to permit equilibration with a gas phase of 8% $CO_2$ in air; the medium was supplemented with heat-inactivated FCS (10% v/v), and contained penicillin (200 $\mu$ ml$^{-1}$) and streptomycin (50 $\mu$g ml$^{-1}$).

Five ml samples of C3H10T$\frac{1}{2}$ cells (200 cells ml$^{-1}$) from subconfluent cultures were distributed among 25 cm$^2$ tissue culture flasks (Falcon) which were incubated overnight at 37°C with caps screwed on lightly to allow for equilibration of the gas phase. Twenty-four hours after plating the cultures were treated with suspensions of Oregon erionite (autoclaved dry, suspended in DMEM and sonicated just prior to addition); as a positive control benzo(a)pyrene was dissolved in acetone and added to the cultures to give a concentration of 1.0 $\mu$g ml$^{-1}$ (final concentration of acetone $<$0.5%).

The cultures were left for 48 h at 37°C after which time a medium change was made. The medium was then changed twice weekly until the cells reached confluence, thereafter the concentration of serum was reduced to 5% and medium changes made weekly. After 6 weeks the cultures were fixed in buffered formalin (10%) stained in methylene blue (1%) and scored for type III transformed foci using the criteria described in Reznikoff *et al*., 1973*b*. Only type III foci were scored as cells from these colonies have been reported as being reliably tumourigenic in syngeneic animals (*Ibid*).

*Unscheduled DNA repair*

The methods used were based on that described by Martin *et al*. (1978), except that exposure to the various test and control substances was for 24 rather than 2.5 h.

(a) *Autoradiographic method* C3H10T$\frac{1}{2}$ cells were grown in 5 cm petri dishes containing sterile 20 mm diameter cover slips. The medium and incubation conditions being as described above. When the cultures were ~80% confluent the medium was replaced with arginine-free MEM (Flow Laboratories Ltd., Irvine, Scotland) supplemented with heat-inactivated dialysed FCS (5% v/v) and reincubated for 24 h at 37°C in an atmosphere of 5% $CO_2$ in air. The medium was then replaced with fresh arginine free MEM and the incubation continued for a further 48 h. At the end of this period hydroxyurea was added to each of the cultures (final conc. 2.5 mM) followed 60 min later by 6-[$^3$H]-dT (21 Ci mM$^{-1}$) giving a final concentration of 10 $\mu$Ci ml$^{-1}$ samples of erionite or a positive control (nitroquinoline-N-oxide NQO) were added to the cultures which were reincubated. Twenty-four hours later the cover slips were removed, washed in PBS, fixed in methanol/acetic acid (3:1), stained in 2% aceto-orcein and processed for autoradiography using Kodak AR10 stripping film. After 14 days storage at −60°C the slides were developed by standard procedures. Each coverslip was examined using a 100 × variable oil immersion objective and silver grains counted automatically using a colony counter (Micromeasurements Ltd.) with its TV camera attached to the microscope. The counting frame was adjusted to correspond to an area of 140 $\mu$m$^2$ and counts were made only when the counting frame was totally enclosed within the outline of a nucleus. Fifty nuclei were counted from each culture and the results of all the replicates for each treatment are reported. Counts were also made on background (non-nuclear) areas, to provide a comparison with the nuclear counts.

(b) *Scintillometric method* C3H10T$\frac{1}{2}$ cells were grown in 25 cm$^2$ tissue culture flasks as described above; A549 cells (Lieber *et al*., 1976) were grown under similar conditions. Treatments with arginine-free medium, hydroxyrea, [$^3$H]-dT and the various agents were as described for coverslip cultures above. Twenty-four hours following treatment the cells were lysed by freezing and thawing the monolayers; the resulting suspension was collected onto cellulose acetate filters and the DNA solubilized as described by Bolognesi *et al*. (1981). The DNA released from the filter was quantified fluorimetrically using Hoechst 33258 and the method of Cesarone *et al*. (1979). Samples of the DNA solution were dissolved in scintillation cocktail and counted in an Intertechnique SL4200 scintillation counter using on-line quench correction.

### Results

*Transformation assay*

The results are presented in Table III and show that exposure to erionite caused an increase in the number of transformed foci as compared to the

**Table III** The effect of Oregon erionite on transformation of C3H10T½ cells

| *Treatment* | | *Survival % control* | *No. of flasks with type III foci / Total no. of flasks* | *Mean no. of type III foci per flask* | *s.e.* |
|---|---|---|---|---|---|
| Oregon erionite | 20 $\mu g\,ml^{-1}$ | 46 | 4/20 | 0.6 | 0.36 |
| Oregon erionite | 10 $\mu g\,ml^{-1}$ | 86 | 1/20 | 0.05 | 0.05 |
| Oregon erionite | 5 $\mu g\,ml^{-1}$ | 95 | 0/20 | 0 | 0 |
| B(a)P | 1 $\mu g\,ml^{-1}$ | 43 | 11/20 | 1 | 0.28 |
| Control | | 100 | 0/20 | 0 | 0 |
| Oregon erionite | 30 $\mu g\,ml^{-1}$ | 39 | 3/12 | 0.3 | 0.19 |
| Oregon erionite | 25 $\mu g\,ml^{-1}$ | 37 | 1/12 | 0.25 | 0.25 |
| Oregon erionite | 20 $\mu g\,ml^{-1}$ | 60 | 6/12 | 0.83 | 0.30 |
| Oregon erionite | 15 $\mu g\,ml^{-1}$ | 66 | 3/12 | 0.5 | 0.30 |
| B(a)P | 1 $\mu g\,ml^{-1}$ | 73 | 5/17 | 1.1 | 0.60 |
| Control | | 100 | 0/30 | 0 | 0 |

B(a)P = Benzo(a)pyrene.

The results reported in this Table are from 2 experiments differing only in the concentrations of erionite used.

negative control cultures. In both experiments the dust caused the appearance of transformed foci when added at concentrations greater than 10 $\mu g\,ml^{-1}$ which may be considered to demonstrate a positive effect.

### *Unscheduled DNA synthesis*

Using the autoradiographic method it was found that erionite caused a significant increase in nuclear labelling at concentrations of 100, 150 and 200 $\mu g\,ml^{-1}$ (Table IV, Figures 2 and 3). As is commonly the case this positive effect diminished and disappeared at higher concentrations of the dust, presumably as a result of cytotoxicity. (Martin *et al.*, 1978).

All the cells in the NQO treatment groups contained labelled nuclei while there was a considerable variation in labelling in the erionite cultures (Table IV, Figure 2). This variability of labelling in the dust exposed cultures is almost certainly due to the fact that the cells came into contact with particles of differing size, shape and probably chemical composition. Thus, while cells in NQO treated cultures received an homogeneous exposure, erionite-treated cultures contained cells which had received a range of insults dependant upon which particles they had encountered.

This range of responses makes it imperative that a representative sample of cells are counted, however, using the autoradiographic technique it was difficult to score the nuclei of cells containing dust especially those in which the nucleus itself was partially obscured by the dust particles. Since this made it impossible to count a truly random

**Figure 2** Frequency distribution of silver grain counts over the nuclei in cells from: (a) a no-treatment control culture; (b) a nithroquinoline-N-oxide (3 $\mu g\,ml^{-1}$) treated culture; (c) an erionite (150 $\mu g\,ml^{-1}$) treated culture. C3H10T½ cultures were processed, treated and UDS estimated as described in Materials and methods.

selection of nuclei, UDS was also measured in both C3H10T½ and in A549 cells using a scintillometric technique which effectively integrates the response of a large number of cells. The results from these experiments confirmed that fibrous erionite can act as an inducer of unscheduled DNA synthesis (Table V).

**Figure 3** Unscheduled incorporation of [$^3$H]-thymidine into C3H10T$\frac{1}{2}$ cells exposed *in vitro* to Oregon erionite (150 $\mu$g ml$^{-1}$) (magnification $\times$775).

**Table IV** Autoradiographic measurement of the unscheduled DNA synthesis in C3H10T½ cells

| *Treatment* | *conc (μg/ml)* | *Mean number of silver grains (Mean ±s.d.) over nuclei* | | *over background* | | *% nuclei with significant labelling* |
|---|---|---|---|---|---|---|
| | | 6.1 | 3.6 | 3.3 | 1.7 | 4 |
| Control | | 5.2 | 3.2 | 2.3 | 0.5 | 4 |
| | | 2.4 | 1.0 | 2.1 | 0.6 | 0 |
| NQO | 3.0 | 30.3 | 12.0 | 1.9 | 0.7 | 100 |
| | | 50.3 | 15.9 | 3.2 | 0.7 | 100 |
| | | 63.4 | 15.1 | 1.6 | 0.7 | 100 |
| Erionite | 25 | 3.7 | 2.9 | 1.9 | 0.6 | 2 |
| | | 2.4 | 2.2 | 1.5 | 0.7 | 2 |
| | | 4.3 | 3.3 | 3.2 | 0.9 | 4 |
| Erionite | 50 | 2.5 | 2.0 | 1.9 | 0.3 | 0 |
| | | 2.7 | 2.3 | 1.3 | 0.5 | 0 |
| | | 3.9 | 5.7 | 1.9 | 0.3 | 6 |
| Erionite | 100 | 35.4 | 46.5 | 2.9 | 1.5 | 56 |
| | | 11.5 | 11.5 | 1.8 | 0.9 | 34 |
| | | 14.4 | 13.7 | 4.0 | 3.6 | 34 |
| Erionite | 150 | 14.3 | 18.1 | 4.4 | 1.3 | 22 |
| | | 18.8 | 13.8 | 3.9 | 2.0 | 54 |
| | | 21.2 | 17.1 | 4.8 | 3.8 | 60 |
| Erionite | 200 | 13.7 | 9.6 | 3.9 | 1.2 | 34 |
| | | 14.6 | 11.6 | 9.6 | 4.7 | 18 |
| Erionite | 250 | 7.6 | 14.5 | 2.9 | 0.6 | 8 |
| | | 6.1 | 2.6 | 3.6 | 0.9 | 0 |
| | | 5.0 | 2.3 | 3.3 | 1.1 | 2 |
| Erionite | 500 | 4.2 | 2.4 | 2.1 | 0.8 | 4 |
| | | 3.0 | 2.1 | 2.0 | 1.1 | 0 |
| | | 1.9 | 1.3 | 1.6 | 1.4 | |

NQO = nitroquinoline N-oxide.

The number of silver grains in a 140 $\mu m^2$ circle over nuclear and other areas was determined as described in the text. Nuclei with >10 grains above the background for the same slide were considered to be significantly labelled and the proportions of such nuclei are given in the last column.

**Table V** Scintillometric measurement of the stimulation of UDS in C3H10T½ and A549 cells exposed to erionite

| *Treatment* | *Specific activity of DNA (dpm ng$^{-1}$ DNA) mean±s.d. C3H10T½ cells* | *A549 cells* |
|---|---|---|
| Control | 1.16 0.29 | 0.68 0.24 |
| NQO $10^{-6}$ M | 1.90 0.02 | N/D |
| NQO $10^{-5}$ M | N/D | 6.72 3.72 |
| Erionite 50 μg ml$^{-1}$ | 2.38 0.24 | 1.66 0.77 |
| 100 μg ml$^{-1}$ | 3.28 0.55 | 1.59 0.19 |
| 200 μg ml$^{-1}$ | 2.97 0.66 | 1.69 0.51 |

N/D = not done.
NQO = nitroquinoline-n-oxide.

The levels of dust required to induce measurable UDS were much higher than those concentrations causing morphological transformation (see Tables III, IV and V). As erionite was shown to be cytotoxic for cells at the lower concentrations i.e. $LC_{50}$ ~20 μg ml$^{-1}$ (average from 2 experiments) the use of higher concentrations in the UDS assays—from 50–500 μg ml$^{-1}$—would suggest that many cells would not survive such treatment. A microscopical examination of the monolayers in the UDS experiments, even at the highest concentrations, showed the cells to be morphologically intact with little or no stripping of cells from the confluent monolayer. The inhibition of DNA repair at the higher dust concentrations would, however, suggest that the erionite was exerting a cytopathic effect and it is doubtful if such cells would be able to undergo cell division. This apparent variation in cytotoxic response in the 2 test systems is most probably due to the different conditions of exposure. In the transformation assays cultures were treated at low cell density and exposure of the actively dividing cells was continued for 14 days before survival was estimated. In contrast the UDS assays involved exposing confluent cultures to erionite for only 24 h.

### Discussion

There are formidable difficulties in choosing appropriate *in vitro* test systems for examination of particulate materials and the systems used in this study have been selected with these difficulties in mind. While there are many transformation systems available the C3H10T½ system was selected because it is not based on subtle changes in colony morphology and has been used to detect many chemical and physical carcinogens (Jones *et al.*, 1976; Benedict *et al.*, 1979; Chan & Little, 1976; Terzaghi & Little, 1976). One important consideration is that this test takes place on the base of the culture vessel where cells and particulates may interact; in those systems using soft agar suspensions the cells and dusts may not have intimate contact, or if contact were made then the particulate material in the agar could provide anchorage points for the growth of normal (non-transformed) cells. The personal experience of the authors and published reports (Daniel & Dehnel, 1980; O'Donovan, 1982) have caused this laboratory to terminate all work with the BHK21 transformation assay (Styles, 1977).

The use of DNA repair assays for the detection of carcinogenic/mutagenic agents has been reviewed recently (Larsen *et al.*, 1982) and these have been advocated by many investigators as a useful screen for the detection of genotoxic agents (San & Stich,

1975; Martin *et al.*, 1978; Yager & Miller, 1978; Martin & McDermid 1981). In this laboratory we have found that both autoradiographic and scintillometric methods must be used with particulates in order to avoid false negative results caused either by adsorption of the DNA on to the dust, or the obscuring of the nuclei by dust particles.

Another fundamental difficulty in examining the activities of particulate materials is in the selection of suitable positive and negative controls. In the systems used in this paper the non-tumourigenic particulates silica, titanium dioxide and non-fibrous derivatives of asbestos all produced negative results (unpublished observations). There are, however, difficulties in regarding these as true negative controls since they are non-cytotoxic to these cell lines and thus may not interact with the cells in any meaningful way. For these reasons it was considered that they could be replaced by no-treatment controls in further experiments. It was clear from these studies that the mere presence of particulate material did not produce spurious positive results. There are no tumourigenic particulates whose mode of action is known which could be used as a suitable positive control.

Many inorganic dusts have been shown to be carcinogenic in experimental animals (Wagner *et al.*, 1980; Wagner, 1982) and it has been suggested that a correlation exists between the pathogenicity of a dust *in vivo* and its cytotoxicity *in vitro* (Brown *et al.*, 1979; Wagner *et al.*, 1982). Other *in vitro* tests especially designed to detect genotoxicity have, however, generally given more variable results. Positive results in chromosomal aberration studies (Sincock & Seabright, 1975), point mutation (Huang *et al.*, 1978) and sister chromatid exchange analysis (Livingston *et al.*, 1980) have suggested that such bioassays may be suitable for the screening of potentially carcinogenic dusts. Other investigators have, however, reported negative results in the sister chromatid exchange assays (Price-Jones *et al.*, 1980) and bacterial mutation tests (Chamberlain & Tarmy, 1977). Examination of crocidolite and amosite asbestos in this laboratory has shown that such agents do not cause either mutation (unpublished observation) or the morphological transformation of C3H10T$\frac{1}{2}$ cells (Poole *et al.*, 1983) though they have proven weakly positive in DNA repair assays (in preparation).

It has been proposed that the pathogenic effect of mineral dusts is mainly attributable to the size and shape of the fibres; those longer than 8 $\mu$m and less than 1.5 $\mu$m diameter are believed to be responsible for the tumourgenicity of these agents (Stanton *et al.*, 1977). Examination of the size distribution of the Oregon erionite used in these studies showed there to be approximately 150 fibres per microgram of dust in this "pathogenic" size range whereas the UICC sample of crocidolite has $1.6 \times 10^5$ such fibres in the same weight (Brown *et al.*, 1978). Thus the number of fibres in the "active" size range would suggest that crocidolite should be many times more active than erionite which it is not. Either the fibre size hypothesis is incorrect, or there is some other property of the zeolite fibre which is responsible for its activities or which augments the activity of the few fibres in the "active" size tange.

These considerations and the positive *in vitro* results reported above make it possible that erionite has qualitatively different activities to those possessed by other mineral fibres. At the very least erionite is quantitatively more active *in vitro* than other pathogenic fibrous dusts. Whilst the extrapolation from *in vitro* to *in vivo* activities is difficult these results are consistent with the demonstration that erionite is a very active carcinogen in both mice (Suzuki *et al.*, 1980) and rats (Wagner, 1982). Indeed it has been reported that it is the "most potent known experimental carcinogenic agent for the pleural mesothelium" (Maltoni *et al.*, 1982).

Recent (unpublished) work in this laboratory had demonstrated that exposure of cultures of C3H10T$\frac{1}{2}$ and A549 cells to fibrous dusts results in increased production of malonaldehyde which is frequently used as an indication of lipid peroxidation caused by free radical reactions (reviewed by Fantone & Ward, 1982). It is possible that the adsorptive and catalytic properties of the erionite could induce free radical chain reactions which differ from those caused by other mineral dusts; erionite formed radicals could be more active in causing cellular and sub-cellular damage. It is also possible that the erionite from both Turkey and Oregon is naturally contaminated with some carcinogenic agent(s) and the fibrous morphology of some particles could transport and hold these unknown agents at vulnerable sites in cultured cells, in intact animals and in humans.

Attempts to obtain the zeolite fibres from the village of Karain in sufficient quantity to enable *in vitro* study are continuing. Meanwhile, our findings that fibrous erionite from a different geographical source can act in ways similar to many conventional carcinogens supports the hypothesis that exposure to this mineral is the cause of the pleural tumours in Turkey. The *in vitro* and *in vivo* activities of this material being such that exposure to other agents need not be invoked as an explanation of the epidemiological findings.

The authors wish to acknowledge the help and advice of Dr. J.C. Wagner and Ms J. Bolan for typing the manuscript.

### References

BARIS, Y.I., SARACCI, R., SIMONATO, L., SKIDMORE, J.W. & ARTVINLI, M. (1981). Malignant mesothelioma and radiological chest abnormalities in two villages in Central Turkey. *Lancet*, **i**, 984.

BENEDICT, W.F., BANGERJEE, A., GARDNER, A. & JONES, P.A. (1979). Indication of morphological transformation in mouse C3H10T$\frac{1}{2}$ Cl 8 cells and chromosomal damage in hamster A(T1) Cl-3 cells by cancer chemotherapeutic agents. *Cancer Res.*, **37**, 2202.

BOLOGNESI, C., CESARONE, C.F. & SANTI, L. (1981). Evaluation of DNA damage by alkaline elution technique after *in vivo* treatment with aromatic amines. *Carcinogenesis*, **2**, 2365.

BROWN, R.C., CHAMBERLAIN, M., GRIFFITHS, M. & TIMBRELL, V. (1978). The effect of fibre size on the *in vitro* biological activity of three types of amphibole asbestos. *Int. J. Cancer*, **22**, 721.

BROWN, R.C., CHAMBERLAIN, M., DAVIES, R., GAFFEN, J. & SKIDMORE, J.W. (1979). *In vitro* biological effects of glass fibres. *J. Environ. Pathol. Toxicol.*, **2**, 1369.

BROWN, R.C., CHAMBERLAIN, M., DAVIES, R. & SUTTON, G.T. (1980). The *in vitro* activities of pathogenic mineral dusts. *Toxicology*, **17**, 143.

CHAN, G.L. & LITTLE, J.B. (1976). Induction of oncogenic transformation *in vitro* by ultraviolet light. *Nature*, **254**, 442.

CESARONE, C.F., BOLOGNESI, C. & SANTI, L. (1979). Improved micro-fluometric DNA determination in biological material using Hoechst 33258. *Anal. Biochem.*, **100**, 188.

CHAMBERLAIN, M. & TARMY, E.M. (1977). Asbestos and glass fibres in bacterial mutation tests. *Mutat. Res.*, **43**, 159.

CHAMBERLAIN, M. (1982). The influence of mineral dusts on metabolic co-operation between mammalian cells in tissue culture. *Carcinogenesis*, **1**, 337.

DANIEL, M.R. & DEHNEL, J.M. (1980). Factors affecting the performance of the Styles cell transformation test. *Carcinogenesis*, **1**, 657.

FANTONE, J.C. & WARD, P.A. (1982). Role of oxygen-derived free radicals and metabolites in leukocyte dependent inflammatory reactions. *Am. J. Pathol.*, **107**, 397.

HUANG, S.L., GAGGIORO, D., MICHELMAN, H. & MALLING, H.V. (1978). Genetic effects of crocidolite asbestos in chinese hamster lung cells. *Mutat. Res.*, **57**, 225.

JONES, P.A., LANG, W.E., GARDNER, A., NYE, C.A., FINK, L.M. & BENEDICT, W.F. (1976). *In vitro* correlates of transformation in C3H10T$\frac{1}{2}$ clone 8 mouse cells. *Cancer Res.*, **36**, 2863.

LARSEN, K.H., BRASH, D., CLEAVER, J.E. & 4 others (1982). DNA repair assays as tests for environmental mutagens. A report of the U.S. EPA Gene-Tox Program. *Mutat. Res.*, **98**, 265.

LIEBER, M., SMITH, S., SZAKAL, A., NEILSON-REES, W. & TODARO, G. (1976). A continuous tumour-cell line from a human lung carcinoma with properties of type II alveolar epithelial cells. *Int. J. Cancer*, **17**, 62.

LIVINGSTONE, G.K., ROM, W.N. & MORRIS, M.V. (1980). Asbestos-induced sister chromatid exchanges in cultured Chinese hamster ovarian fibroblast cells. *J. Environ. Pathol., Toxicol.*, **4-2**, 372.

MALTONI, C., MINARDI, F. & MORISI, L. (1982). The relevance of the experimental approach in the assessment of the oncogenic risks from fibrous and non-fibrous particles. The ongoing project of the Bologna Institute of Oncology. *Med. Lavoro.*, **73**, 393.

MARTIN, C.N., McDERMID, A.C. & GARNER, C.R. (1978). Testing of known carcinogens and non-carcinogen for their ability to induce unscheduled DNA synthesis in Hela cells. *Cancer Res.*, **38**, 2621.

MARTIN, C.N. & McDERMID, A.C. (1981). Testing of 42 coded compounds from unscheduled DNA synthesis using Hela cells in culture. In *International Program for the Evaluation of Short-Term Tests for Carcinogenicity* (Eds. Ashby & de Serres) Amsterdam: Elsevier-North Holland, p. 207.

O'DONOVAN, M.R. (1982). Some factors influencing the behaviour of BHK 21 Cl 13 cells in soft agar medium. *Carcinogenesis*, **3**, 961.

POOLE, A., BROWN, R.C. & FLEMMING, G.T.A. A study in the cell transforming ability of amosite and crocidolite asbestos. *Environ. Health Perspec.*, (In press).

PRICE-JONES, M., GUBBINGS, G. & CHAMBERLAIN, M. (1980). The genetic effects of crocidolite asbestos. Comparison of chromosome abnormalities and sister exchanges. *Mutat. Res.*, **79**, 331.

REZNIKOFF, C.A., BRANKOW, D.W. & HEIDELBERGER, C. (1973*a*). Establishment and characterization of a cloned line of C3H mouse embryo cells sensitive to post confluence inhibition of division. *Cancer Res.*, **33**, 3231.

REZNIKOFF, C.A., BERTRAM, J.S., BRANKOW, D.W. & HEIDELBERGER, C. (1973*b*). Quantitative studies of chemical transformation of cloned C3H mouse embryo cells sensitive to post confluence inhibition of cell division. *Cancer Res.*, **33**, 3239.

ROHL, A.N. LANGER, A.M., MONCURE, G., SELIKOFF, I.J. & FISCHBEIN, A. (1982). Endemix pleural disease associated with exposure to mixed fibrous dust in Turkey. *Science*, **216**, 518.

SAN, R.H.C. & STICH, H.K. (1975). DNA repair synthesis of cultured human cells; a rapid bioassay for chemical carcinogens. *Int. J. Cancer*, **16**, 284.

SINCOCK, A. & SEABRIGHT, M. (1975). Induction of chromosomal changes in Chinese hamster cells by exposure to asbestos dusts. *Nature*, **258**, 56.

STANTON, M.F., LAYARD, M., TEGERIS, A., MILLER, E., MAY, M. & KENT, E. (1977). Carcinogenicity of fibrous glass: pleural response in the rat relationship to fibre dimension. *J. Natl Cancer Inst.*, **58**, 587.

STYLES, J.A. (1977). A method for detecting carcinogenic organic chemicals using mammalian cells in culture. *Br. J. Cancer*, **36**, 558.

SUZUKI, Y., ROHL, A.N., LANGER, A.M. & SELIKOFF, I.J. (1980). Mesothelioma following intraperitoneal administration of zeolite. *Fed. Proc. Fed. Am. Soc. Exp. Biol.*, **39**, 3 (Abstr.).

TERZAGHI, M. & LITTLE, J.B. (1976). Oncogenic transformation *in vitro* after split dose X-irradiation. *Int. J. Radiat. Biol.*, **29**, 583.

WAGNER, J.C., BERRY, G. & POOLEY, F.D. (1980). Carcinogenesis and mineral fibres. *Br. Med. Bull.*, **36**, 53.

WAGNER, J.C. (1980). Environmental and occupational exposure to natural mineral fibres. In *Biological Effects of Mineral Fibres*. (Ed. Wagner) Lyon: I.A.R.C., p. 995.

WAGNER, J.C., CHAMBERLAIN, M., BROWN, R.C. & 4 others. (1982). Biological effects of tremolite. *Br. J. Cancer*, **45**, 352.

WAGNER, J.C. (1982). Health hazards of substitutes: In *World Symposium on Asbestos*, Quebec. May, 1982.

YAGER, J.D. & MILLER, J.A. (1978). DNA repair in primary cultures of rat hepatocytes. *Cancer Res.*, **38**, 4385.